# Surgical resection of recurrent differentiated thyroid cancer: patterns, detection, staging, and treatment of 683 patients

**DOI:** 10.3389/fendo.2023.1301620

**Published:** 2023-12-06

**Authors:** Nan Liang, Han Zhang, Chengqiu Sui, Rui Du, Canxiao Li, Jingting Li, Gianlorenzo Dionigi, Daqi Zhang, Hui Sun

**Affiliations:** ^1^ Division of Thyroid Surgery, The China-Japan Union Hospital of Jilin University, Jilin Provincial Key Laboratory of Surgical Translational Medicine, Jilin Provincial Precision Medicine Laboratory of Molecular Biology and Translational Medicine on Differentiated Thyroid Carcinoma, Changchun, Jilin, China; ^2^ Division of General Surgery, The First Affiliated Hospital of Dalian Medical University, Dalian, Liaoning, China; ^3^ Department of Medical Biotechnology and Translational Medicine, Division of General and Endocrine Surgery, Istituto Auxologico Italiano IRCCS, University of Milan, Milan, Italy

**Keywords:** thyroid cancer, recurrence, surgery, morbidity, second surgery

## Abstract

**Background:**

Despite improvements in overall survival, the recurrence of differentiated thyroid cancer (DTC) is becoming more common and remains a challenge necessitating accurate reappraisal of the patient. This study aimed to describe the characteristics, reasons, morbidity, and strategies of second operations for DTC.

**Methods:**

This was a retrospective observational study of patients with DTC who underwent a second surgery between June 2008 and June 2021 in the Department of Thyroid Surgery at China–Japanese Union Hospital, Jilin University, P.R. China. All clinical characteristics were recorded, and the analysis was estimated using SPSS.

**Results:**

Second surgeries were detected in 683 patients. The proportion of second operations changed with the update of international guidelines from 2015 (*P* < 0.001). The true recurrence rate progressively increased from 21.3% to 61.5%. The rate of an “absence of preoperative FNA” or an “absence of intraoperative pathology at first surgery” decreased from 49.8% to 12.7%, while that of a “misdiagnosis of preoperative FNA at second surgery” decreased from 10% to 1.8%. The most common tumor location during the second surgery was the lateral lymph nodes (n = 104, 36.5%), with a median time to relapse of 36 months. Completion of thyroidectomy and lymph node dissection correlated with the second operation.

**Conclusion:**

After 2015, second surgeries were more standardized, the incidence of complications decreased, and real recurrence became the most common reason for a second DTC surgery.

## 1 Introduction

The incidence of thyroid cancer has been increasing all over the world (1). Differentiated thyroid cancer (DTC) is the principal histologic type of thyroid gland cancer, accounting for the majority of thyroid malignancies (2). In addition, outcomes related to various treatment options can vary significantly based on different clinicopathologic factors, tumor size, and genetic variants related to tumor biology. Regardless of etiology and treatment strategy, DTC can recur (3). As such, postoperative surveillance is warranted, and when recurrence is detected, the reassessment of disease status and treatment options is necessary. Within this context, the incidence of second DTC surgeries has also increased (3).

Treatment algorithms and data on the management of recurrent DTC are critical to guide clinical decisions. Due to the advances in early diagnosis, improvements in guidelines, and the optimization of treatment options, patients with recurrent DTC now have better prognoses (3). Furthermore, due to altered anatomy, the risk of complications such as hypoparathyroidism and vocal fold paralysis following a second surgery is higher than that after the initial surgery (4). Moreover, with the further improvement of DTC guidelines and the standardization of diagnosis and treatment, the reasons for DTC second surgery have also changed. Meanwhile, the diagnosis and treatment strategy of DTC second surgery are affected by many factors (5). The effective and standard treatments are worthy studied (6). In this context, further investigation of the epidemiologic, treatment, and clinical features of recurrent DTC is essential. This study aims to clarify the status of the second surgeries by analyzing the features, reasons and surgical strategies of the second surgery of DTC patients in our center, so as to provide a theoretical basis for the standardized treatment of the second surgeries.

## 2 Materials and methods

### 2.1 Design study

This was a retrospective observational study.

### 2.2 Setting

This single-center study was carried out at the China–Japan Union Hospital of Jilin University, China.

### 2.3 Time frame

This study lasted for 13 years, i.e., from June 2008 to June 2021.

### 2.4 Ethics

The institutional ethics committee of the China–Japan Union Hospital of Jilin University approved this study (20220506023). All included patients signed informed consent forms.

### 2.5 Eligibility criteria

Patients of either sex were included if they had a cytological diagnosis of DTC, had experienced just one thyroid surgery procedure before, and were indicated to undergo a redo surgery, and the second operation was offered at the Thyroid Surgery Division of the China–Japan Union Hospital of Jilin University.

### 2.6 Exclusion criteria

Patients with benign histology, undetermined malignant potential tumors, medullary thyroid cancer, non-thyroidal malignancies, incomplete information, or previous tumor ablation were excluded. We also did not include any individual who had received prior anti-tumor treatment, including chemotherapy, radiotherapy, or immunotherapy, or who was participating in an interventional trial with an expected impact on the outcome of the present study. Finally, any patient with a contraindication to redo surgery; who was not available for follow-up; who did not undergo primary tumor resection at initial surgery, i.e., only experienced local incision or palliative treatment; who did not provide informed consent.

### 2.7 Definition of recurrent disease

According to Duren ‘s criteria, relapses of thyroidal carcinoma were classified as either local (may present itself in the residual thyroid lobe or in the thyroid bed where surgery was performed), loco-regional (may present in the cervical lymph nodes of the central compartment or lateral-cervical nodes), or distant (distant metastasis) more than one year after the previous operation (7).

### 2.8 Definition of persistent disease

Persistent disease was defined by a positive Tg result, an abnormal neck ultrasound image, or persistently increased levels of TgAb.

### 2.9 Definition of disease-free status

Patients were considered disease-free when they showed (1) no clinical evidence of a tumor, (2) no evidence of a tumor on radioactive iodine imaging and/or cervical ultrasound, or (3) an unstimulated serum Tg concentration of <0.2 ng/mL or stimulated Tg concentration of <1 ng/mL in the absence of interfering antibodies (Abs) (8).

### 2.10 Detection of recurrent diseases

All included patients underwent thyroid function tests (including serum T3, T4, and TSH; Tg; and TgAbs), neck ultrasound (US) imaging, preoperative fine-needle aspiration cytology, FNA-Tg (if suspected LNM), subsequently postoperative pathology analyses. Some patients took the CT scan, MRI, PET when necessary (8).

### 2.11 Pathological analysis

During the second surgery, we routinely obtained tumor tissue samples for histological analysis by surgical resection. Recruited DTC patients were divided into papillary thyroid cancer (PTC) and follicular thyroid cancer (FTC) groups.

### 2.12 Preoperative redo-surgery evaluations

Before their second surgery, each patient underwent a new laryngoscopy examination to determine the condition of their laryngeal recurrent nerve. In addition, iPTH, calcitonin, and blood calcium tests were routinely performed.

### 2.13 Postoperative assessments and follow-up

The follow-up for patients after the second surgery is similar to that following the primary surgery; specifically, US imaging was performed, and biochemical indicators (e.g., Tg; serum T3, T4, and TSH; and blood calcium) were measured.

### 2.14 Data collection

**Figure 1:**
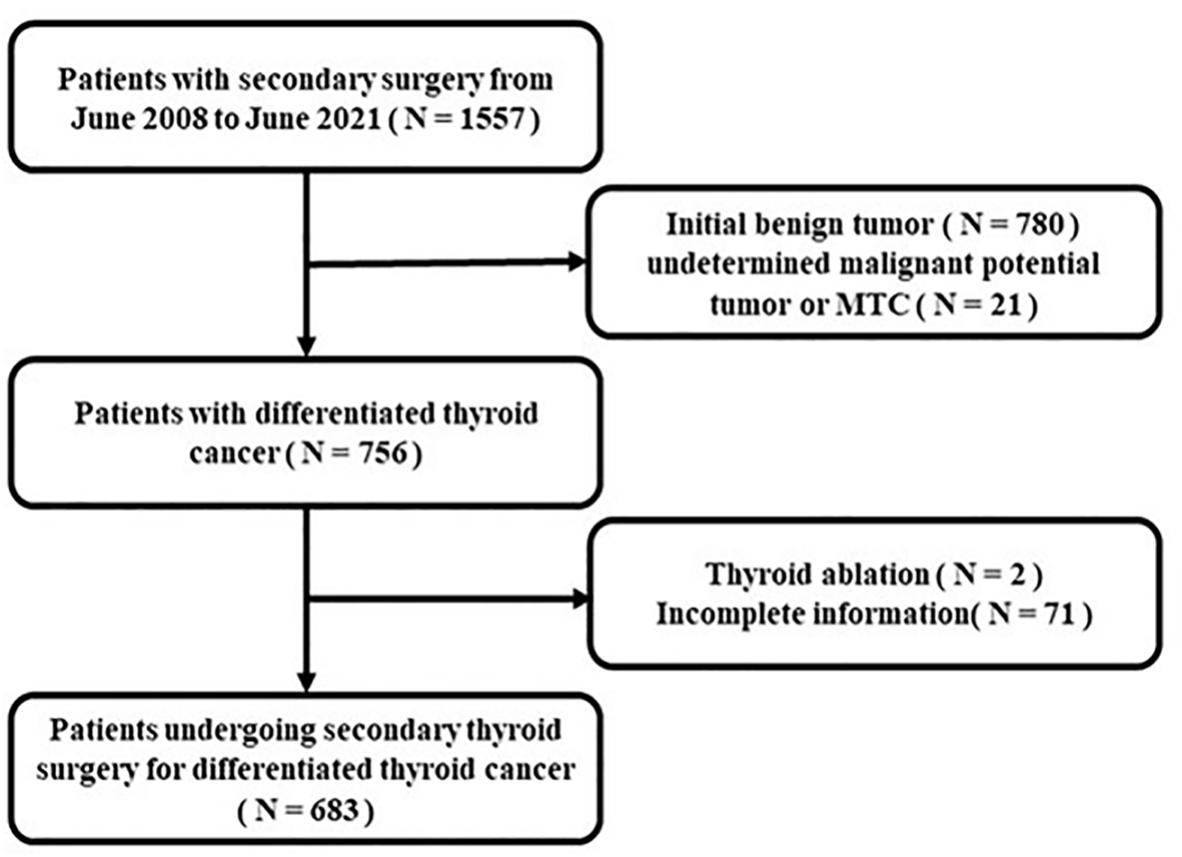
Flowchart of the inclusion and exclusion criteria.

A total of 1557 patients undergoing re-operation were identified. After applying the exclusion criteria, 683 (43.8%) patients were included in this study (**Figure 1**).

#### 2.15 Initial approach and surgery for recurrent disease

Thyroid: Total thyroidectomy (TT); near TT; sub-TT; lobectomy + isthmusectomy; <lobectomy (i.e., nodulectomy) (9).

Lymph node: central lymph node dissection (CLND); unilateral lateral lymph node dissection (LLND); CLND +unilateral LLND; bilateral LLND; CLND + bilateral LLND (9).

### 2.16 Clinical outcomes measured

We collected and reviewed the initial pathology using the patients’ medical history. The reasons for the second surgery were classified into five groups and also divided into objective reasons, subjective reasons, and real recurrence. Objective reasons included “lack of preoperative FNA or intraoperative pathology in the initial surgery” and “lack or misdiagnosis of preoperative FNA in the second surgery.” Subjective reasons included “less-experienced surgeons” and a “less-experienced paramedical team.” The time of recurrence was measured from the date of the previous neck operation.

### 2.17 Time trends comparison

We segmented the timing of reoccurrence into three parts according to the 2012 Chinese expert consensus and 2015 ATA guidelines, as follows: June 2008 to December 2012, January 2013 to December 2015, and January 2016 to June 2021 (8).

### 2.18 Statistical analysis

We analyzed all data using SPSS version 23.0 (IBM Corporation, Armonk, NY, USA). All results are shown as mean ± standard deviation, median, and interquartile range or count (percentage) values. Continuous variables were analyzed by Fisher’s exact test and the rank-sum test. Categorical variables were analyzed with an analysis of variance test, or, in the case of non-normal distribution, using the Kruskal–Wallis test. *P* < 0.05 was considered statistically significant. Charts were generated using GraphPad Prism version 8.0 (GraphPad Software, San Diego, CA, USA).

## 3 Results

### 3.1 Clinical, pathological, and surgical features

A total of 683 patients were enrolled, comprising 172 men (25.2%) and 511 women (74.8%). The male-to-female ratio was 1:2.97 (**Table 1**). Patients’ mean age at the initial and second surgeries was 37.94 ± 11.39 and 40.74 ± 11.25 years, respectively. The median body mass index was 24.2 (21.8; 26.7). The majority of tumors were PTC (97.2%), with just 2.8% being FTC. Total thyroidectomy was the most common operation among both initial surgeries (n = 222, 32.5%) and second surgeries (n = 317, 46.4%). As for cervical lymph node dissection during the second surgery, CLND + unilateral LLND was performed most often (n = 252, 36.9%). The median operative time for the second operation was 180 (135; 232) min. Thirty-six patients (5.3%) developed vocal cord paralysis, and 73 subjects (10.7%) had transient hypoparathyroidism.

**Table 1:** Clinical features of second-surgery DTC patients.

Features	N(%)	Features	N(%)
**Total**	683		
**Sex**		**The length of secondary surgery(min)**	180(135;232)
Male	172(25.2%)	**Vocal code motion disorder**	
Female	511(74.8%)	Caused by initial surgery	51(7.5%)
**Age of initial diagnosis**	37.94 ± 11.39	Unilateral	51
<55	634(92.8%)	Bilateral	–
>=55	49(7.2%)	Caused by second surgery	36(5.3%)
**Age of secondary surgery**	40.74 ± 11.25	Unilateral	36
<55	608(89.0%)	Bilateral	–
>=55	75(11.0%)	**Parathyroid function after secondary surgery**	
**BMI**	24.2(21.8;26.7)	Normal	610(89.3%)
**Type of primary tumor**		Hypoparathyroidism	73(10.7%)
PTC	664(97.2%)	**Reasons of secondary surgery**	
FTC	19(2.8%)	Lack of preoperative FNA or intraoperative pathology in the initial surgery	190(27.8%)
**Initial thyroid surgery**		Lack or misdiagnosis of preoperative FNA in the second surgery	28(4.1%)
<lobectomy	143(20.9%)	Less-experienced surgeons	95(13.9%)
lobectomy + isthmusectomy	130(19.0%)	Less-experienced of the paramedical team	85(12.4%)
Sub-total thyroidectomy	63(9.2%)	Recurrence	285(41.7%)
Near-total thyroidectomy	125(18.3%)	**Second surgery year**	
Total thyroidectomy	222(32.5%)	2008.06-2012.12	211(30.9%)
**Second thyroid surgery**		2013.01-2015.12	189(27.7%)
Total thyroidectomy	317(46.4%)	2016.01-2021.06	283(41.4%)
Near-total thyroidectomy	124(18.2%)		
lobectomy + isthmusectomy	20(2.9%)		
Total thyroidectomy(initial)	222(32.5%)		
**Second lymph node dissection**			
No lymph node dissection	10(1.5%)		
CLND	99(14.5%)		
Unilateral LLND	134(19.6%)		
CLND + Unilateral LLND	252(36.9%)		
Bilateral LLND	76(11.1%)		
CLND + Bilateral LLND	112(16.4%)		

### 3.2 Persistent vs. recurrent disease

54.1% of patients who underwent re-operation suffered from persistent disease, and 41.7% of patients experienced true recurrence after an initial disease-free interval.

### 3.3 Reasons for the second surgery

Every patient’s clinical information was critically reviewed thoroughly, and the specific inferred the reasons were analyzed and classified. Objective reasons included “lack of preoperative FNA or intraoperative pathology in the initial surgery” (n = 188, 27.5%) and “lack or misdiagnosis of preoperative FNA in the second surgery” (n = 19, 2.8%). Subjective reasons included “less-experienced surgeons” (n = 99, 14.5%) and a “less-experienced paramedical team” (n = 92, 13.5%). In fact, the “less-experienced surgeons” refer to improper surgical procedures during the initial operation, and similarly, the so-called “low experienced paramedical team” refer to the incomplete or inaccurate evaluation results during the initial evaluation. A total of 285 patients (41.7%) experienced “real recurrence.”

### 3.4 Modeling time trends

Further analysis found that, with the continuous updating of guidelines (**Table 2**), the proportion of different reasons for the second surgery changed (*P* < 0.001). The percentage of patients with true recurrence gradually increased from 21.3% (first stage) to 34.9% (second stage) and 61.5% (third stage), while the percentage of second surgeries performed due to objective reasons gradually decreased (*P* < 0.001), with operations attributed to a “lack of preoperative FNA or intraoperative pathology in the initial surgery” decreasing from 49.8% in the first stage to 12.7% in the third stage and those attributed to a “lack or misdiagnosis of preoperative FNA in the second surgery” dropping from 10% in the first stage to 1.8% in the third stage. However, there was no significant difference in the proportion of operations performed for subjective reasons among the three stages. **Supplementary Table 1** shows the differences in clinicopathological characteristics of patients who underwent second surgeries in each of the three stages. Total thyroidectomy (*P* < 0.001) became the main option for the second thyroid surgery. The length of the second surgery was also gradually shortened (225 vs. 180 vs. 150 min, *P* < 0.001).

**Table 2:** Different reasons of second-surgery DTC patients in different stages.

	2008.06-2012.12	2013.01-2015.12	2016.01-2021.6	*P*
Reasons of secondary surgery				<0.001
Lack of preoperative FNA or intraoperative pathology in the initial surgery	**105(49.8%)**	**49(25.9%)**	**36(12.7%)**	
Lack or misdiagnosis of preoperative FNA in the second surgery	**21(10.0%)**	**2(1.1%)**	**5(1.8%)**	
Less-experienced surgeons	**24(11.4%)**	**34(18.0%)**	**37(13.1%)**	
Less-experienced of the paramedical team	**16(7.6%)**	**38(20.1%)**	**31(11.0%)**	
Recurrence	**45(21.3%)**	**66(34.9%)**	**174(61.5%)**	

**Figure 2:**
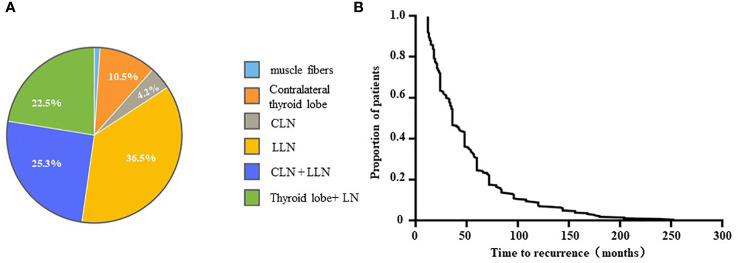
The tumor location of secondary surgery **(A)** and the recurrence time curve **(B)**.

We compiled the tumor location from the second surgery in cases of real recurrence (**Figure 2A**); the main tumor location during second surgeries was the lateral lymph nodes (n = 104, 36.5%), followed by the central + lateral lymph nodes (n = 72, 25.3%) and the thyroid lobe + lymph node (n = 64, 22.5%). In addition, there were 30 patients with recurrence in the contralateral thyroid lobe, accounting for 10.5% of all cases. We further studied the recurrence time of DTC patients among 285 cases with real recurrence (**Figure 2B**). More than 50% of the cohort experienced relapse within 36 months, and the majority of recurrence cases (71.5%) occurred within the first 5 years after the initial surgery. The follow-up duration ranges from 12 months to 264 months, with a median re-operation time of 36 months.

### 3.5 Second surgery strategies

The reason for the second surgery is an important factor in the selection of second surgical procedures. **Table 3** shows that in cases re-treated due to a “l Lack of preoperative FNA or intraoperative pathology in the initial surgery,” total thyroidectomy (n = 102, 54.3%) and near-total thyroidectomy (n = 74, 39.4%) each accounted for half of the second thyroid surgeries. CLND + unilateral LLND was the routine second lymph node dissection procedure (n = 104, 55.3%). In case re-treated due to a “Lack or misdiagnosis of preoperative FNA in the second surgery”, near-total thyroidectomy (n = 12, 63.2%) was the main type of second thyroid surgery. Among cases re-treated due to “less-experienced surgeons” or a “less-experienced paramedical team,” total thyroidectomy was the most common type of second thyroid surgery. However, there were some differences in CLND procedures during second lymph node dissection for two reasons: first, the “less-experienced paramedical team” group mostly performed lateral lymph node dissection; second, among patients with real recurrence, total thyroidectomy (n = 259,91.1%) was the major type of second thyroid surgery. The majority second lymph node dissection types were CLND + unilateral LLND (n = 98, 34.4%) and unilateral LLND (n = 75, 26.3%).

**Table 3:** Different surgical strategy in “second surgery” reasons of DTC.

	Lack of preoperative FNA or intraoperative pathology in the initial surgery	Lack or misdiagnosis of preoperative FNA in the second surgery	Less- experienced surgeons	Less- experienced of the paramedical team	Recurrence
**Initial thyroid surgery**	188(100%)	19(100%)	99(100%)	92(100%)	285(100%)
<lobectomy	137(72.9%)	4(21.1%)	2(2.0%)	1(1.1%)	0
lobectomy + isthmusectomy	0	2(10.5%)	14(14.1%)	17(18.5%)	97(34.0%)
Sub-total thyroidectomy	47(25.0%)	7(36.8%)	5(5.0%)	3(3.3%)	0
Near-total thyroidectomy	4(2.1%)	6 (31.6%)	19(19.2%)	26(28.3%)	70(24.6%)
Total thyroidectomy	0	0	59(59.6%)	45(48.9%)	118(41.4%)
**Second thyroid surgery**					
Total thyroidectomy	102(54.3%)	6(31.6%)	29(29.3%)	39(42.4%)	141(49.7%)
Near-total thyroidectomy	74(39.4%)	12(63.2%)	10(10.1%)	7(7.6%)	21(7.4%)
lobectomy + isthmusectomy	12(6.4%)	1(5.3%)	1(1.0%)	1(1.1%)	5(1.8%)
Total thyroidectomy(initial)	0	0	59(59.6%)	45(48.9%)	118(41.4)
**Second lymph node dissection**					
No lymph node dissection	3(1.6%)	5(26.3%)	0	1(1.1%)	1(0.4%)
CLND	36(19.1%)	1(5.3%)	6(6.0%)	11(12.0%)	45(15.8%)
Unilateral LLND	8(4.3%)	5(26.3%)	20(20.2%)	25(27.2%)	75(26.3%)
CLND + Unilateral LLND	104(55.3%)	4(21.1%)	32 (32.3%)	15(16.3%)	98(34.4%)
Bilateral LLND	5(2.7%)	3(15.8%)	14(14.1%)	26(28.3%)	28(9.8%)
CLND + Bilateral LLND	32(17.0%)	1(5.3%)	27(27.3%)	14(15.2%)	38(13.3%)

## 4 Discussion

The rate of second surgeries for thyroid cancer is increasing year by year, and the recurrence rate of well-DTC has reached 30% (10, 11). Radioactive iodine has been an important adjuvant treatment for persistent/recurrent DTC, but it may not be able to improve the prognosis very well (12, 13). Therefore, it is essential to have a standard second surgery for DTC at hand (14). This study enrolled all patients who experienced second surgeries for DTC at a single center over 13 years to summarize the re-operation reasons, strategies, and development trends among them.

In addition to recurrence, reasons for a second surgery among DTC patients also include persistent diseases and both subjective and objective factors. Bates et al. (15) indicated that about 77.2% of second surgeries may be attributed to persistent disease rather than true disease recurrence. Similarly, we found that, before the release of the Chinese expert consensus in 2012, the proportion of second surgeries performed due to subjective and objective reasons at our center was about 78.7%. However, with the improvement of the guidelines, real recurrence (61.5%) became the most common reason for second surgeries among DTC patients at our center, especially after the release of the 2015 guideline, and the percentage of second surgeries performed for subjective and objective reasons has decreased to 38.5%. This also means that real recurrence has become the main reason for second surgeries at this stage, and the difficulty of the second surgery has also increased. In our true recurrence group, lateral cervical lymph nodes (36.5%) were the main tumor sites during the second surgery, followed by the central + lateral cervical lymph nodes (26.3%) and the thyroid lobe + lymph nodes (22.5%), while contralateral thyroid lobe recurrence accounted for 10% of cases. The results reported by Xu et al. (16) differ slightly from ours. They pointed out that, in 232 patients with persistent/recurrent DTC, 43.1% had lesions in the lymph nodes, 17.7% had lesions in the remnant thyroid or thyroid bed, and 39.2% had lesions in both locations. This difference may be due to a slight variation in the study methods and populations, but both our study and that by Xu et al. each pointed out that the second operation focuses more on lymph node dissection, which increases the difficulty of the operation.

In terms of complications, Medas et al. (17) observed that, in 152 patients with second surgeries for benign or malignant recurrent diseases, the rates of permanent hypoparathyroidism (10%) and transient RLN injury (4.6%) were greater than those following the initial surgery. A 2009 review (18) reported rates of 9.5% and 6.4% for permanent hypoparathyroidism and vocal fold disease, respectively, with both being significantly higher than those following initial thyroidectomy. Michael P et al. (19) reported a 31% rate of inadvertent partial or complete parathyroid resection. Similarly, in our center, patients with hypoparathyroidism and vocal code motion disorders totaled 10.7% and 5.3% of the population after second surgeries, respectively. After 2015, the proportion of patients with postoperative hypoparathyroidism declined to 7.4%. This phenomenon suggests that our surgical technique improved after the 2015 ATA guidelines were published.

In patients with DTC, the decision about the method of secondary surgery is mainly determined by US, computed tomography, and MRI (20, 21). However, the choice of second surgery modality is closely related to the reason for the second surgery (22) and is also typically inseparable from the cause of the initial surgery. Therefore, we need to conduct in-depth analyses of the causes and auxiliary medical examinations of patients and then decide on the parameters of the second surgery (23).

## 5 Limitations of the study

There were several limitations in this study. First, though this was a single center and retrospective study, it was a relative large cohort. In addition, since majority of those patients were taken the initial surgery at some other hospitals. It was difficult to obtain the complete information about initial surgery, such as pathological subtype, tumor invasion, high-volume lymph node metastasis, et al. Finally, there was no further follow up information for after the second surgery.

## 6 Conclusions

Significant changes have occurred in the features of DTC patients undergoing second surgeries with updates to the guidelines. The reasons for the second surgery and the procedure of the initial surgery are key factors determining the strategy of the second surgery among DTC patients. With the development of the surgical standard, skill proficiency, and intraoperative nerve monitoring (IONM) technology improvements, the proportion of second surgeries attributed to subjective and objective reasons declined. In the new era, real recurrence became the primary reason for second surgeries for DTC. We look forward to more accurate and unified guidelines for DTC second surgeries.

## Data availability statement

The raw data supporting the conclusions of this article will be made available by the authors, without undue reservation.

## Ethics statement

The institutional ethics committee of the China–Japan Union Hospital of Jilin University approved this study (20220506023). The studies were conducted in accordance with the local legislation and institutional requirements. Written informed consent for participation in this study was provided by the participants’ legal guardians/next of kin. Written informed consent was obtained from the individual(s), and minor(s)’ legal guardian/next of kin, for the publication of any potentially identifiable images or data included in this article.

## Author contributions

NL: Writing – review & editing, Conceptualization, Data curation, Funding acquisition, Methodology, Supervision. HZ: Writing – original draft, Conceptualization, Data curation, Formal Analysis, Methodology. CS: Resources, Writing – review & editing. RD: Resources, Writing – review & editing. CL: Resources, Writing – review & editing. JL: Resources, Writing – review & editing. GD: Writing – review & editing, Methodology, Supervision, Visualization. DZ: Writing – review & editing, Conceptualization, Methodology, Resources, Supervision. HS: Writing – review & editing, Conceptualization, Data curation, Funding acquisition, Project administration, Resources, Supervision, Visualization.
