# Supplementary material for: Surgical resection of recurrent differentiated thyroid cancer: patterns, detection, staging, and treatment of 683 patients
Source: Front Endocrinol (Lausanne). 2023 Dec 6;14:1301620. doi: 10.3389/fendo.2023.1301620 (PMC10731281; doi:10.3389/fendo.2023.1301620)
Supplement: Supplementary file 1 [file DataSheet_1.pdf]

Supplementary table1. Differences of clinical features of second-surgery DTC patients in different stages

	2008.6—2012.12	2013.01-2015.12	2016.01—2021.6	*P*
**Sex**				0.716
Male	49(23.2%)	48(25.4%)	75(26.5%)	
Female	162(76.8%)	141(74.6%)	208(73.5%)	
**Age of initial diagnosis**	38(29;46)	38(31;45)	37(30;45)	0.721
<55	195(92.4%)	177(93.7%)	262(92.6%)	0.902
>=55	16(7.6%)	12(6.3%)	21(7.4%)	
**Age of secondary surgery**	40(30;47)	41(33;47)	41(33;49)	0.117
<55	189(89.6%)	172(91.0%)	247(87.3%)	0.454
>=55	22(10.4%)	17(9.0%)	36(12.7%)	
**Second thyroid surgery**				＜0.001
Total thyroidectomy	80(43.0%)	106(87.6%)	131(85.1%)	
Near-total thyroidectomy	104(55.9%)	11(9.1%)	9(5.8%)	
lobectomy + isthmusectomy	2(1.1%)	4(3.3%)	14(9.1%)	
Total thyroidectomy(initial)	-	-	-	
**Second lymph node dissection**				＜0.001
No lymph node dissection	6(2.8%)	1(0.5%)	3(1.1%)	
CLND	6(2.8%)	22(11.6)	71(25.1%)	
Unilateral LLND	39(18.5%)	22(11.6%)	72(25.4%)	
CLND + Unilateral LLND	5(45.0%)	69(36.5%)	89(31.4%)	
Bilateral LLND	21(10.0%)	25(13.8%)	29(10.2%)	
CLND + Bilateral LLND	44(20.9%)	49(25.9%)	19(6.7%)	
**The length of secondary surgery(min)**	225(185;290）	180(140;230)	150(100;190)	＜0.001
